# The emergence of sex differences in primary pain during adolescence: a conceptual developmentally-oriented biopsychosocial model and opportunities for further investigation

**DOI:** 10.1186/s12887-025-06283-3

**Published:** 2025-11-04

**Authors:** Hannah Hagy, Esmeralda Hidalgo-Lopez, Christel Portengen, Alexis Holman, Andrew Schrepf, Daniel J. Clauw, Steven E. Harte, Adriene M. Beltz, Amy M. Bohnert, Chelsea M. Kaplan

**Affiliations:** 1 Department of Psychology, Edward Hines, Jr. VA Hospital, Hines, IL USA; 2 Chronic Pain and Fatigue Research Center, Department of Anesthesiology, University of Michigan Medical School, 24 Frank Lloyd Wright Dr., Lobby M, PO Box 385, Ann Arbor, MI 48105 USA; 3 Department of Psychology, University of Michigan, Ann Arbor, MI USA; 4 Department of Internal Medicine, University of Michigan, Ann Arbor, MI USA; 5 Neuroscience Graduate Program, University of Michigan, Ann Arbor, MI USA; 6 Department of Psychology, Loyola University Chicago, Chicago, IL USA

**Keywords:** Chronic pain, Primary pain, Adolescent development, Sex differences, Neuroimaging, Puberty

## Abstract

Adolescence begins with puberty and is characterized by striking hormonal, physical, psychosocial and neurobiological changes. In childhood, male and female youth have a similar prevalence of chronic pain conditions and similar pain processing. However, this changes dramatically during puberty when females become more pain sensitive and develop significantly more chronic pain compared to males. This pattern persists throughout the lifespan, with female adults twice as likely to report chronic widespread pain as males. The type of pain that seems to increase in prevalence during adolescence (especially in females) is now termed primary pain, meaning that the pain is the primary problem (not secondary to tissue damage or inflammation), and is likely due to nociplastic pain mechanisms. This review takes a developmentally-oriented biopsychosocial perspective on the emergence of sex and gender differences in primary pain by highlighting adolescence as a pivotal period marked by pubertal maturation. Drawing from larger literatures on pediatric pain, neuroimaging, and adolescent development, we identify key biological, behavioral, cognitive-affective, and sociocultural factors that may influence the emergence of sex or gender differences in pain, focusing on primary pain. We then make recommendations for future studies, highlighting the unique insights that can be garnered by considering sex and gender differences and puberty in pain research to inform precision treatments at the most critical developmental periods.

## Introduction

Sex and gender matter for pain: female adults experience higher rates of most chronic pain conditions, including fibromyalgia, migraine/headache, temporomandibular disorder, irritable bowel syndrome, and chronic pelvic pain relative to male adults [1]. These conditions are collectively referred to as chronic overlapping pain conditions or, in clinical medicine, as primary pain conditions, emphasizing that pain is the primary problem and not a consequence of another disease [2, 3]. The mechanistic term, *nociplastic*, has been recently introduced to describe pain that results from altered nociception despite the absence of peripheral pathology [4, 5]. Sensitization within the central nervous system (CNS) is a key driving factor of nociplastic mechanisms in primary pain [6]. Individuals with primary pain often have multisite pain, multimodal sensory sensitivity, fatigue, sleep problems, dyscognition and mood disturbances, further highlighting the role of the CNS in these conditions [6–8]. Genetic predisposition, potentially via genes associated with brain structure and function [9, 10], dysregulated immune function, peripheral sensitization and psychosocial factors may also contribute to the development of primary pain [6]. 

Nociplastic processes can also superimpose and amplify peripherally-mediated pain, leading to a female predominance in pain conditions typically conceptualized as *nociceptive* (i.e., pain due to tissue damage or inflammation) [11]. For example, female adults report higher rates of painful osteoarthritis compared to males [12]. Importantly, this is not because females have more peripheral pathology or nociceptive input – in fact, the correlation between self-reported pain and radiographic evidence is poor in both sexes [13, 14]. Even in individuals without chronic pain, females are generally more pain and sensory sensitive than males [1, 15]. 

Sex differences in primary pain emerge during puberty, which marks the beginning of adolescence. In early childhood, males and females have a similar prevalence of chronic primary pain conditions and no reliable differences in experimental pain sensitivity [1, 16]. This changes around age 12 (which is the average age of menarche for females, with the maturation of males following females by a couple years), when females become more sensitive to painful stimuli and their rates of primary pain begin to rise dramatically [1, 16, 17]. A large meta-analysis found that 24.5% of female youth with an average age of 13.4 years reported chronic multisite pain, compared to 15.1% of males [18]. Despite the high prevalance and enormous impacts of primary pain on children’s lives [19–22] and its lasting consequences into adulthood [22–27], relatively little attention has been given to the underlying mechanisms driving these sex differences.

**Fig. 1:**
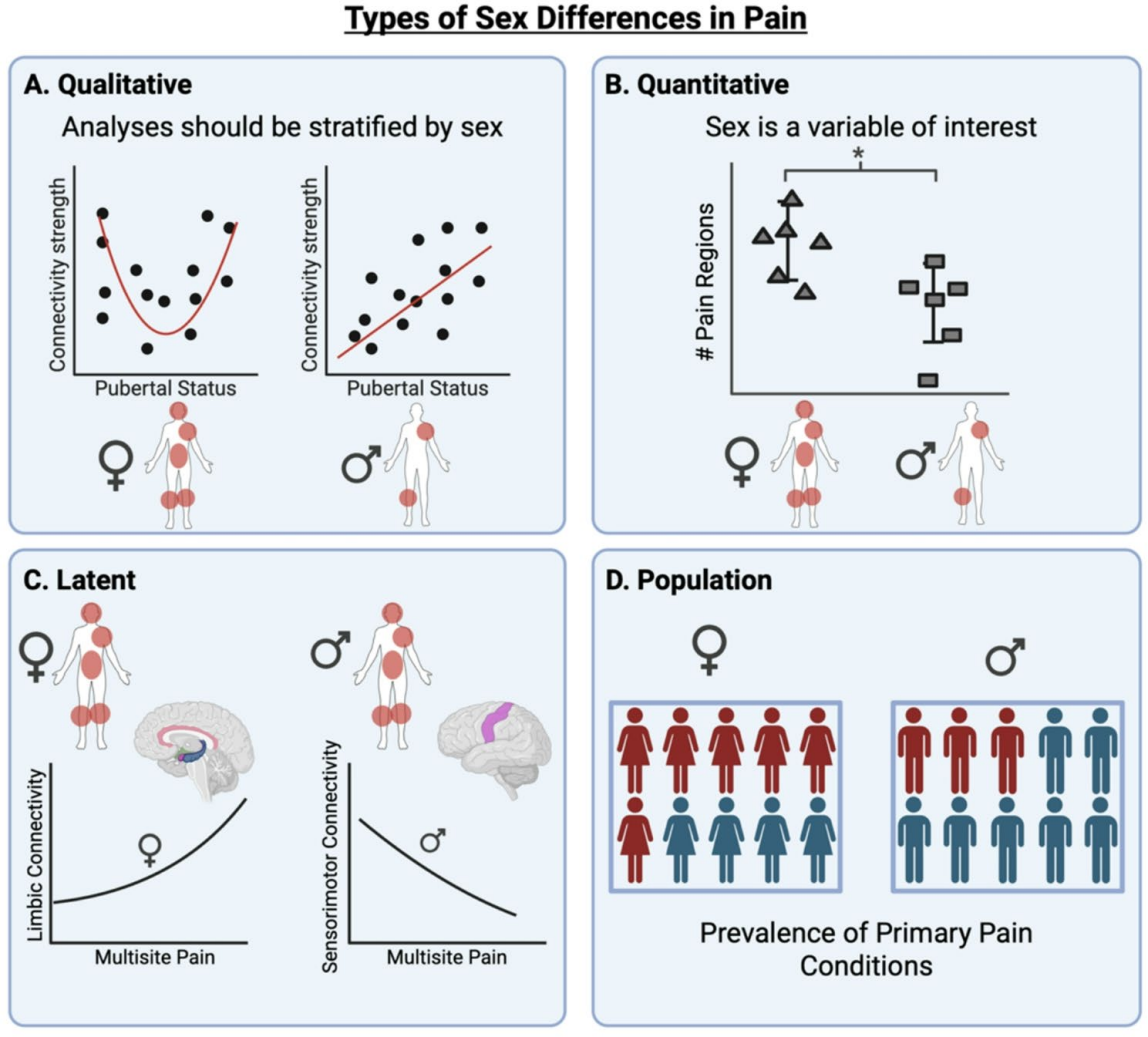
Although some sex differences in pain are well established, others are likely missed or misrepresented, as different types of sex differences – that require different analysis strategies – are seldom examined. Types of sex differences are not mutually exclusive. In qualitative sex differences (**A**) there are unique processes in males and females (such as pubertal development and its relation with neural network connectivity underlying pain) and therefore analyses should be sex-stratified. In quantitative sex differences (**B**), the same process is present in males and females but to different degrees (e.g., on average, females have more painful body regions relative to males), so sex can be a variable of interest in analyses. Latent sex differences (**C**) relect different underlying mechanisms in males and females. For example, both males and females may have multisite pain, although the neural correlates may be unique for each sex. Finally, population-based sex differences (**D**) reflect prevalence rates that vary by sex, such as increased rates of primary pain conditions in females compared to males. For latent and population sex differences, optimal analysis techniques may vary with the research question and data sets, requiring sex-stratified approaches and/or the inclusion of sex as a variable of interest (see [33] for more discussion and guidance on analysis strategies)

The National Institutes of Health’s ‘sex as a biological variable’ policy [28] increased awareness of the importance of including males and females in research [29], however, it is still somewhat rare for studies to explicitly examine sex differences or sex-mediated and -moderated effects [30, 31]. For example, most studies with chronic pain patients use single-sex cohorts or mixed-sex cohorts and statistically control for sex, overlooking the issue of sex effects altogether [32]. Although it can be challenging to consider sex in study design a priori, overlooking sex, either by including sex as a “confound” or averaging across males and females *a posteriori* could lead to results that do not apply to anyone (who is average-sexed?) or could lead to spurious results if males and females have different underlying pain processes that are essentially cancelled out in analyses. Instead, novel insights into primary pain could be revealed by thoughtfully mapping sex differences onto research questions and analyses, as differences may not only be quantitative in nature (reflecting the same process to different degrees in males and females, permitting linear sex-based comparisons). Sex differences may also be qualitative (reflecting different processes in males and females), latent (reflecting different underlying mechanisms in males and females), or population-based (reflecting prevalence rates that vary by sex) – all of which may require separate statistical models for males and females [33, 34]. This is illustrated in Fig. 1.

Gender also plays an important role in shaping pain experiences [35]. Gender is a multifaceted construct that reflects the sociocultural expectations regarding roles, behaviors and identities deemed appropriate for females and males [36]. Although sex and gender are distinct concepts, disentangling their roles in the development of primary pain is difficult [35] and the relative contributions of biological and sociocultural processes are unique to each individual. Because of the neurobiological focus of this paper, sex differences are largely highlighted, but gender-related factors and language are used when describing the psychosocial markers of primary pain. We acknowledge that both sex and gender are not binary but exist on spectra. Since previous studies have predominantly operationalized sex and gender as binary constructs, this review will use corresponding language (male/female, boy/girl) to make that distinction [36], noting that research concerning gender-expansive individuals or sex as a continuum is lacking.

The pubertal emergence of sex and gender differences in chronic pain is likely due to a multitude of overlapping and intertwined factors, as suggested by prior reviews [29, 32, 37–42]. The aim of this paper is to build on these prior reviews by emphasizing the most developmentally relevant factors that may increase risk for *primary* pain in females with a particular focus on underlying brain architecture. Examining pain onset within this developmental framework may be useful to understand *why* sex and gender matter.

### A developmentally-oriented biopsychosocial perspective on the emergence of sex/gender differences in primary pain

**Fig. 2:**
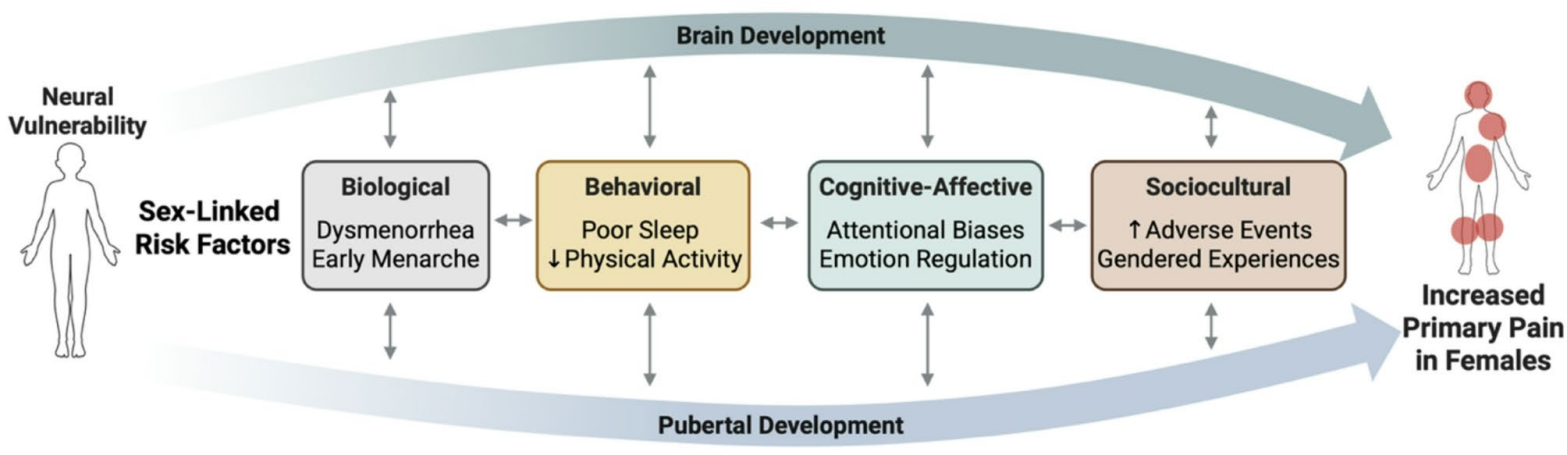
Sex differences in primary pain emerge during adolescence, a pivotal period marked by pubertal maturation and brain development, however the exact mechanisms underlying these sex differences remain unknown. We hypothesize that the function of certain brain networks may be key susceptibility factors, which in concert with several overlapping sex- or gender-linked factors (spanning biological, behavioral, cognitive-affective and sociocultural domains) may explain the greater prevalence of primary pain in females compared to males that emerge during adolescence

Drawing on the existing research on pediatric pain, neuroimaging, and adolescent development, this conceptual review offers a developmentally-oriented biopsychosocial perspective to understand the emergence of sex differences in primary pain in adolescence. This review is not meant to be exhaustive, but rather, to provide a broad overview of biopsychosocial factors that likely contribute to sex and gender disparities in primary pain that emerge during puberty. A central component of this perspective is that the function of certain brain networks may be key susceptibility factors, which in the face of several sex- or gender-linked overlapping factors, including aspects of biological (hormones, menstruation), behavioral (sleep and physical activity), cognitive-affective (attentional biases, emotion regulation), and sociocultural (adverse events, gendered experiences) development may explain the greater rates of primary pain in females that emerge around puberty (see Fig. 2). We acknowledge that chronic pain likely emerges from several different, overlapping pathways (i.e., equifinality), suggesting there is not one factor alone that is enough to explain the sex differences in primary pain for all unique youth.

The second central component concerns pubertal development, which is not only defined by reaching specific milestones (i.e., status), but also by the age when those milestones were reached compared to peers of the same sex (i.e., timing) and the pace of maturation (i.e., tempo) [43]. This review highlights how the CNS and pubertal hormones (adrenal and gonadal) work in concert with other adolescent psychosocial and behavioral changes to drive sex or gender differences, while considering how those differences are conceptualized and analyzed in the limited, extant literature. Finally, we consider how future research can address key gaps about the emergence of sex and gender differences in primary pain during adolescence with a focus on specific pubertal influences on the developing brain.

### Brain structure and function as a susceptibility factor for primary pain

Neuroimaging studies in adults with primary pain conditions consistently show altered brain structure and function in sensory processing regions regardless of sex [6]. There is also extensive evidence of altered functional connectivity within and between brain networks, although the exact nature of these changes is somewhat inconsistent and varies between studies. For example, a recent study using UK Biobank data noted striking decreases in functional connectivity among several brain networks in individuals with primary pain relative to pain-free controls [44], although many other (albeit smaller) studies have found primarily increases in functional connectivity between pro-nociceptive areas of the brain (e.g [45–48]).,. The modest body of existing research indicates that children with primary pain have similar alterations in brain structure and function as those observed in adult populations [38], including altered default mode network (DMN), sensorimotor network (SMN) and salience network (SLN) functional connectivity and activation during experimental pain [49–62]. A recent sex-stratified study using data from the Adolescent Brain and Cognitive Development (ABCD) Study found that while both males and females with multisite pain exhibited reduced within-SMN connectivity, there were sex differences in between-network connectivity, with males uniquely showing greater DMN-SMN connectivity compared to those with no pain [63]. Taken together, although the findings are somewhat heterogeneous and more work is needed to delineate the specific patterns of alterations, it is clear that CNS plasticity (i.e., nociplastic pain) is a hallmark of primary pain.

To date, there has been only one study examining the CNS in children prior to pain onset. Leveraging data from the ABCD Study, this study found increased functional connectivity in brain regions related to pain processing (e.g., insula/salience network, primary somatosensory and motor cortices, cingulate and default mode regions) in children (ages 9–10) one year *before* they developed multisite pain relative to control children who remained pain-free [64]. These findings suggest an underlying CNS vulnerability to primary pain, as these alterations in connectivity were present prior to the development of pain in multiple body regions. Notably, no sex differences in functional connectivity were detected in this study, perhaps because only quantitative sex differences were examined and most males were pre-pubertal. This study suggests that brain function may predispose an individual to developing pain, although additional studies are needed to confirm these findings.

### Biological factors: changes in hormones and the CNS during adolescence

Puberty marks the beginning of adolescence and is driven by the activation of the hypothalamic-pituitary-gonadal axis, which initiates major hormonal shifts. These include increased production of adrenal androgens in both sexes, along with a marked rise in gonadal testosterone in males and larger increases in gonadal progesterone and estradiol in females. Importantly, the CNS triggers the initiation of puberty, and it is also profoundly changed by it.

Some evidence suggests that sex differences in brain structure and function coincide with pubertal development [65], and these differences are at least partially mediated by hormonal mechanisms [66, 67]. Adrenal and gonadal hormones play a role in reorganizing brain regions relevant for pain processing [68–70], which may differentially affect pain vulnerability across sexes [37]. However, methodological inconsistencies – such as limited use of sex-stratified analyses – and a possible non-linear interaction between sex and puberty (when only quantitative sex differences are considered) limit the likelihood of capturing sex-specific developmental trajectories [67]. 

For example, cerebral blood flow in the insula and DMN, regions with altered connectivity in adults with multisite pain [47, 71–73], varies with pubertal status in ways dependent on sex [74]. Males exhibit a linear decline over time, whereas females show a U-shaped curve, with higher cerebral blood flow in early and advanced puberty compared to mid-puberty. Similarly, subcortical structures such as the amygdala, a region critical for pain modulation, follow sex-related developmental trajectories: amygdala volume increases during puberty, with males displaying a gradual and steady increase, but females showing an inverted U-shaped curve, having pronounced increases in volume during early puberty followed by a subsequent decline [75]. These qualitative sex differences were only revealed through sex-stratified analyses [74]. Moreover, a longitudinal investigation by Nguyen and colleagues go beyond sex, focusing on sex-related hormones [76]. They found that testosterone and dehydroepiandrosterone (DHEA) levels moderated the structural covariance between the amygdala and cortical regions involved in the sensory and affective dimensions of pain as well as in cognitive control (e.g., somatosensory, parietal and medial prefrontal cortex) [76]. Lower levels of these hormones were associated with positive amygdala-cortical connections, whereas higher levels were linked to negative connections. Overall, these findings suggest that puberty is a sensitive period in which gonadal hormones dynamically influence the remodelling of brain regions (including those involved in pain processing), and underscore the need for sex-stratified, longitudinal, and hormone-informed research to understand neurodevelopmental contributions to sex differences in primary pain.

Given these relations among puberty, hormones and the brain, it is expected that hormones also influence pain modulation directly. Emerging evidence indicates that both adrenal androgens (e.g., DHEA) that contribute to puberty-related body hair growth (e.g., underarm and pubic) and gonadal androgens (e.g., testosterone)play a sex-related role in pain [77–79]. Also, testosterone appears to exert antinociceptive effects in adult men and women [80–82]. For example, adult women with fibromyalgia have lower testosterone levels compared to healthy individuals [83], and day-to-day fluctuations in testosterone and progesterone are negatively related to pain severity, while there is no relation with estradiol [84]. Meanwhile, studies on pregnancy, menopause, the menstrual cycle and hormone manipulation, both in animals and humans, show considerable variability in both the direction and magnitude of the findings regarding pain sensitivity and ovarian hormonal levels, likely owing to variability in study design and methods [1, 85, 86]. 

Most studies have examined the relations between adrenal and gonadal hormones and pain in adults, but combined with findings in animal studies, a similar pattern is expected for human adolescents. Although research is sparse, a recent study by Li and colleagues utilizing ABCD data found that advanced pubertal status at ages 9–10 was associated with greater risk of pain onset one year later [87]. However, higher testosterone and DHEA levels were associated with a lower risk of future pain incidence and intensity only in males [87]. In population-based longitudinal [88, 89] and cross-sectional studies [90–92] studies of youth with back pain there is a positive relation between pubertal development and reported back pain, regardless of age (ranging from 11 to 17 years), and this was found for male and female youth. It remains unknown if or how these findings relate to the development of brain structure and function. Moreover, studies examining the effects on adrenal androgens on primary pain are warranted, given its effects on brain organization [93] and potential relations to mental health [94–96] through the hypothalamus-pituitary-adrenal axis.

One hormone-related biological factor that is unique to females is menarche. For some, menstruation involves the experience of recurrent pain (referred to as dysmenorrhea). Conceptually, dysmenorrhea can be due to an entirely peripheral source (e.g., heavy menstrual flow), but like other nociceptive pain conditions, it might also be due to superimposed nociplastic mechanisms, such as peripheral or central sensitization [97–100]. An early age at menarche (i.e., early pubertal timing with onset < 12 years old) is associated with higher rates of dysmenorrhea and greater pain severity [101]. Furthermore, females with early menarche are more likely to experience a range of primary pain conditions, including headache, stomachache, musculoskeletal pain, and multisite pain, compared to peers with on-time or late menarche [102–106]. Thus, pubertal timing and the experience of menstruation may be important biological factors to assess when considering the emergence of sex differences in primary pain.

### Behavioral factors

Accumulating evidence suggests there is a bidirectional relation between pain and adolescent behaviors, including sleep and physical activity, with patterns varying by sex [107, 108]. Sleep disturbances appear to be a stronger predictor of chronic pain in adolescents than vice versa [109]. For example, a recent study found that sleep problems predicted new-onset multisite pain in children [110]. When sex and CNS function are incorporated, however, this relation becomes more nuanced: Female, but not male, adolescents with multisite pain reported more sleep problems than those without pain, a relation that was partially explained by reduced within-network connectivity of the SMN [63]. Puberty also matters. Sleep patterns shift during adolescence (e.g., later bedtimes, shorter duration), and this shift occurs earlier in development for females compared to males, likely due to females entering puberty earlier [111]. Notably, in the large, longitudinal AddHealth study, sleep problems increased with advancing pubertal development only for females; however, sleep duration decreased across both sexes with advancing puberty [112]. Girls also experience a 2.75 times increased risk for insomnia following menarche, while there are no sex differences observed prior to this developmental milestone. To date, no study has considered how changes in sleep across the pubertal transition may differentially relate to sex differences in primary pain. Taken together, the literature suggests that sleep difficulties are unique to girls and may be another (latent) factor that confers risk for heightened pain experiences [113]. 

Physical activity promotes health and wellbeing in multiple areas. Research suggests that engaging in regular physical activity can improve functioning in chronic pain populations [114, 115]. Youth with chronic pain often forego physical activity in an effort to avoid risk of injury or further exacerbation of their pain [116]. Activity avoidance also may be a consequence of depression that frequently co-occurs with pediatric pain conditions [117]. Declines in physical activity across the pubertal transition occur across gender; however, the timing and magnitude of the decline in physical activity differs between boys and girls [118–122]. Physical activity diminishes earlier in development for girls than boys, with girls exhibiting lower levels of physical activity throughout adolescence, both of which may confer additional risk for primary pain.

### Cognitive-affective factors

Cognitive-affective factors are also relevant to primary pain in pediatric populations [123–125]. Broadly, research has demonstrated links between pain and cognitive domains, such as attention, memory, processing, executive functioning, and decision making [126]. There are many cognitive-affective domains that show gender differences, but for this conceptual review, we focused on domains that may be particularly relevant across puberty to explain sex-related differences in primary pain. One domain is attentional biases for pain experiences (i.e., hypervigilance); for example, adult women had greater difficulty disengaging from a pain-related fear conditioning stimulus relative to men [127]. Attention increases as adolescents progress through puberty, likely mediated by the prefrontal cortex, and adolescents with early pubertal timing show larger increases in attention skills than their on-time or late-timing peers [128]. There is a lack of research on gender differences in attention and pain, incorporating puberty, in adolescents, though given that attention problems predicted the appearance of new multisite pain one year later in children who were pain-free at baseline, more studies are warranted [110]. 

Research has also demonstrated intricate links between emotion regulation (a multi-faceted process of modifying an emotional experience toward a desired state [129]) and the emergence of gender differences in primary pain. In childhood, related constructs like effortful control show a gender difference favoring girls [130], but processes may change with development, as emotion regulation problems during adolescence are normative and may result from a lack of synchronized maturation of neurobehavioral systems involved in reactivity and regulation [131–134]. Indeed, there is a growing interest in going beyond affect to consider the intersection of emotion regulation and pain. One recent paper suggests that emotion regulation difficulties are a risk factor for chronic pain conditions [129]. Boys and girls also generally employ different emotion regulation strategies, with girls being more likely to ruminate or seek social support than boys [135, 136]. Although it has yet to be examined empirically in adolescents, rumination could contribute to pain experiences in unique ways for boys and girls. For example, Meints and colleagues (2017) found that adult men and women demonstrated no differences in overall pain catastrophizing; however, rumination mediated the relation between gender and differences in experimental pain tolerance and thresholds [137]. More work examining gender-related emotion regulation shifts over puberty is needed [138], focusing on how these shifts may relate to pain experiences in adolescents.

Related to emotion regulation, gender differences in anxiety and depression exist, with gender differences in depression emerging during the pubertal transition with girls, for the first time, exhibiting markedly higher rates of depression relative to boys. Puberty also plays a role in the development and maintenance of internalizing symptoms: Compared to their peers with on-time pubertal development, girls with early timing are at increased risk for developing depression and anxiety, whereas boys with early timing are at increased risk for anxiety and boys with late timing are at increased risk for depression [43, 139–141]. Anxiety is also differentially associated with pain experiences in adult men and women, with pain-related anxiety linked to pain severity in men, and anxiety for bodily experiences and fear of pain linked to pain severity in women [142–144]. The developmental origins of pain-related anxiety are currently unclear, but the necessity of considering sex in future investigations on the topic is apparent. Given the links between internalizing problems and pain, it is currently unclear whether and how puberty-linked depression increases and anxiety are related to primary pain, particularly in conjunction with the presence of other factors in our model, but this is a crucial area of inquiry.

### Sociocultural factors

The influence of sociocultural factors in shaping pain and its management is well-documented [145, 146]. Sociocultural factors, including adverse events and gendered experiences, play a particularly crucial role in shaping pain perception and expression [15, 147]. Importantly, early adverse events have been linked to pubertal timing [148–150], demonstrating the influence of sociocultural factors on biological processes relevant to pain. Girls and gender-expansive individuals are more at risk for complex traumas [151] and adverse events [152] which could exacerbate underlying pain risk. One recent study utilizing ABCD data during early adolescence demonstrated significant gender-by-puberty interaction effects on pain outcomes, and concluded that psychological (e.g., internalizing symptoms) and sociocultural factors (e.g., discrimination) may be more relevant for multisite pain compared with regional pain during early adolescence; unfortunately, these factors were not examined separately in boys and girls, precluding inferences about qualitative or latent differences [153]. 

Additionally, sociocultural beliefs and expectations about gender (e.g., femininity and masculinity) can play a central role in shaping pain responses and reports, as pain expression is generally more socially acceptable among women [154, 155]. This can lead to biased reporting of pain, with women being more likely to report pain than men, but also pain being considered less severe when reported by women than men [156]. Men are also less likely to seek medical help for pain, partly influenced by conflict with their perceived gender norms [157]. Also within the family context, caregivers can reinforce pain behaviors among children. In one study, parents paid more attention to their daughters’ pain, whereas they were more likely to distract their sons when they were in pain. Importantly, girls were particularly susceptible to the reinforcing effect of parental attention to pain, resulting in elevated symptom levels among adolescent girls, pointing toward qualitative gender differences in this parental reinforcement [158]. 

### Areas for future research

Future research around the development of primary pain must consider sex and gender as well as puberty. The nature of a sex or gender difference (e.g., quantitative versus qualitative) should determine the analysis strategy, such as whether sex or gender should be included as a variable of interest in a mixed sample or whether analyses should be stratified by sex or gender, respectively. Simply “controlling for” sex or gender is not recommended [33]. It is also critical for future research to measure puberty, specifically defining what aspect of development is being considered (i.e., pubertal status, timing, or tempo) and ensuring the available data are up to the task (e.g., status and timing are often confounded in cross-sectional reports); details regarding the measurement and operationalization of puberty are described in [159]. Notable interindividual differences are evident for both the timing and tempo of puberty. In addition, there are sex differences in these aspects of puberty [160] and they can have different impacts on the developing brain. Future research should carefully consider how these pubertal processes are captured given the multiple ways in which they can be measured [161]. Pubertal status is optimally assessed using Tanner staging [162], based on physical examination, or by self-report based on pictures or line drawings. Status can also be assessed with the Pubertal Development Scale (PDS), a 5-item parent- or self-report questionnaire [66, 163]. Additionally, salivary, urine, or blood hormonal measurement can be used, provided that samples are collected with high fidelity and frequently enough to differentiate intra-individual variation (e.g., diurnal and menstrual cycles) from pubertal development [66]. Likewise, pubertal timing can be assessed using these same subjective (Tanner staging, PDS) or objective measures (biological measures), but requires longitudinal data; in brief, growth curves could be fit to pubertal development trajectories across assessments, and timing (e.g., age at mid-puberty, such as the middle Tanner stage or PDS score) could be abstracted from an individual’s curve [159, 164]. A retrospective measure of timing has recently been validated (against prospective data), and is ideal for use in samples in which participants have already completed their pubertal development [164]. Pubertal tempo also requires longitudinal data (e.g., time between events of puberty, yearly rate of change, or peak rate of change) and ideally captures both the beginning and endpoint of puberty [159, 165]. The optimal methodology depends on the research question of interest and study design [66, 159]. Puberty, however, is often poorly defined and specified using any of these methods in neuroimaging research in particular. Thus, there is a critical need for more studies using valid and reliable puberty measures for assessing not only status, but also timing and tempo, and for analysis plans that consider sex and gender in the context of adolescent primary pain (see Table 1 for examples and general guidelines). A better grasp of the link between pain, sex and gender, pubertal status, and CNS structure and function could enable researchers and clinicians to identify and target modifiable risk and protective factors at this critical point in development.

**Table 1:** Examples of future directions which incorporate sex-stratified, longitudinal, and hormone-informed research designs to understand sex differences in primary pain. Also listed are general guidelines to consider when conducting studies of sex and puberty in adolescents with primary pain

Risk Factor	Example Future Directions		Key Considerations Related to Sex and Puberty
	Sex or Gender Differences	Pubertal Development	
Biological	Which sex-related changes in brain networks confer risk for the development of primary pain in adolescence?	Does pubertal tempo influence the development of dysmenorrhea and future primary pain in female adolescents?	• The type of sex difference (e.g. quantitative versus qualitative) should inform the analysis plan. For example, consider if the analysis should be stratified by sex. See [33] for detailed guidance. • Consider non-linear relationships. • Pubertal status can be assessed with Tanner Staging or the PDS. If using the PDS, the age of the child should be considered when deciding on child or parent report. • Pubertal timing is ideally measured with longitudinal data, but may also be recalled retrospectively. • Pubertal tempo requires longitudinal data which ideally captures the beginning and end of pubertal development. • Hormone assessment (from saliva or serum) should occur at the same time of day and longitudinally to distinguish relatively consistent individual differences from pubertal development.
Behavioral	Do inter-relations among physical activity, brain development, and primary pain vary by sex in adolescence?	In what ways do pubertal hormones relate to sleep disturbances in adolescents with and without primary pain?	
Cognitive-Affective	Are there gender differences in attention difficulties that precede or follow the development of primary pain in adolescence?	Which links between anxiety and pain experiences are mediated or moderated by pubertal status?	
Sociocultural	How do adverse events impact neurobiology and increase risk for primary pain in unique ways for male and female adolescents?	How are the physical changes marking puberty related to the pressure to conform to gender norms, including the reporting of pain?	

*PDS* Pubertal Development Scale

## Conclusion

During adolescence the prevalence of primary pain increases in females relative to males. The mechanisms underlying sex and gender differences in pain remain largely unknown but likely involve the cascade of hormonal changes associated with puberty, which affects all organ systems, including the CNS, and is related to a multitude of behavioral, cognitive-affective, and even sociocultural factors. Adolescence appears to be a second sensitive period for neural reorganization induced by massive changes in the neuroendocrine system. This creates a window of vulnerability for developing primary pain in youth, as highlighted in the presented developmentally-oriented biopsychosocial model. There is a dearth of research linking pain, puberty, and CNS development, but primary pain in youth (and its prevalence in females) surely involves the interplay of these factors.

## Data Availability

Not applicable.

## Abbreviations

CNS: Central nervous system
DMN: Default mode network
SLN: Salience network
SMN: Sensorimotor network
ABCD Study: Adolescent brain cognitive development study
PDS: Pubertal development scale
DHEA: Dehydroepiandrosterone
